# Effectiveness of Integrative Therapy for Parkinson’s Disease Management

**DOI:** 10.3389/fnagi.2019.00040

**Published:** 2019-02-26

**Authors:** Yeonju Woo, Min Kyung Hyun

**Affiliations:** ^1^Department of Preventive Medicine, College of Korean Medicine, Dongguk University Graduate School, Seoul, South Korea; ^2^Department of Preventive Medicine, College of Korean Medicine, Dongguk University, Gyeongju, South Korea

**Keywords:** Parkinson’s disease, integrative medicine, medicine, Korean traditional, complications, patient care

## Abstract

**Objectives:** To investigate the effectiveness of integrative therapy on prevalence and length of hospitalization and management of major complications of Parkinson’s disease (PD) in the South Korea.

**Methods:** This study was a retrospective cohort analysis conducted using the National Health Insurance Service-National Sample Cohort in the South Korea. Patients over 65 years old who were newly diagnosed with PD during 2007–2011 were identified. The integrative therapy group was defined as patients treated with both Korean medicine (KM) and biomedicine, and the monotherapy group consisted of patients treated with biomedicine alone. From PD diagnosis to 2013, the prevalence and annual length of hospitalization because of PD and major complications (dementia, depression and pneumonia/sepsis) were analyzed using logistic regression, ANOVA and *t*-tests after propensity score (PS) matching with a 1:1 ratio.

**Results:** After PS estimation and matching, the cohort used in the analysis included 228 subjects (114 integrative therapy group, 114 monotherapy group). Sex, age, index year, comorbidity, severity of disability, neurologic care, and anti-parkinsonism medication (levodopa, ropinirole, pramipexole, selegiline) were adjusted in both groups. The prevalence of hospitalization due to pneumonia/sepsis was 0.50 times (95% C.I.: 0.26–0.96) lower in the integrative therapy group than the monotherapy group, which was statistically significant (*p* = 0.038). The prevalence and annual length of total hospitalization and hospitalization because of PD, dementia, and depression in the integrative therapy group showed positive results compared to the monotherapy group, but these differences were not statistically significant.

**Conclusion:** It has not been clearly identified that integrative therapy with KM and biomedicine for PD management is better treatment for patients compared to biomedicine monotherapy; however, we found a clue of better result in integrated therapy. Therefore, further investigation by increasing the number of subjects is needed to confirm the findings presented herein.

## Introduction

Parkinson’s disease, which is the second most frequent neurodegenerative disease after Alzheimer’s disease, is caused by an imbalance between the inhibitory action of dopamine and the excitatory action of acetylcholine because of decreases in dopamine (Rizek et al., 2016; Delamarre and Meissner, 2017). The incidence and prevalence of PD increases with age, especially after one reaches their 60s.

Parkinson’s disease patients have motor symptoms including bradykinesia, rigidity, resting tremor, and postural instability; however, non-motor symptoms may also be prominent, including cognitive impairment, depression, and autonomic disturbances (Rogers et al., 2017). Generally, PD is caused by an interaction between genetic and environmental factors (Delamarre and Meissner, 2017). Pesticides, history of melanoma, and traumatic brain injury have also been reported as increased risk factors of PD, but the promotion of physical activity is known to prevent PD (Ascherio and Schwarzschild, 2016).

Levodopa is the gold-standard for treatment of motor symptoms of PD (Rizek et al., 2016). However, PD is progressive and worsens with time, and there is currently no cure; accordingly, it is necessary to treat and care for PD-related symptoms of patients (Elbaz et al., 2016). Therefore, about 40% of PD patients use alternative therapies including acupuncture, tai chi, and herbal medicine to complement their standard treatments (Ghaffari and Kluger, 2014). Indeed, previous studies have shown that herbal formulas, acupuncture and tai chi might be safe and beneficial to PD patients (Bega and Zadikoff, 2014; Cwiekala-Lewis et al., 2017; Lee and Lim, 2017; Noh et al., 2017; Shan et al., 2018). In addition, there have been studies to find out the benefit of using biomedicine and KM concurrently in PD patients (Leem, 2016; Yang et al., 2016; Jang et al., 2018; Lee et al., 2018).

In the present study, the effectiveness of integrative therapy consisting of both biomedicine and KM on the management of PD patients was investigated.

## Materials and Methods

### Data Source

All citizens of the South Korea receive health insurance, and medical treatment and prescription data of all healthcare institutions and pharmacies has been loaded into a national database maintained and managed by the NHIS. The NHIS built the NHIS-NSC database, which consists of representative anonymized data extracted from national health insurance records pertaining to about 1,000,000 members of the total population in 2002 who were followed until 2013. The personal privacy of each participant was protected by de-identification of the data (Lee et al., 2017). The NHIS-NSC 2^nd^ version (2002–2015) of the NHIS-NSC was generated in 2017, but the data masked 2,980 diagnoses, including those of cancer and infective diseases. Therefore, the 2^nd^ version could not be used in this study.

### Study Design

**FIGURE 1:**
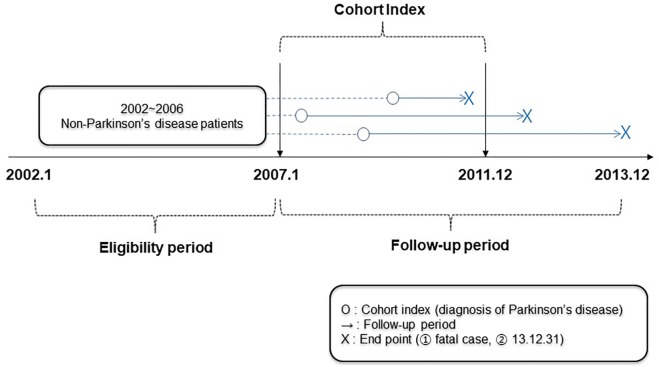
Study design.

This was a retrospective cohort study. The eligibility period was set from January 2002 (the start date of the NHIS-NSC) to December 2006, while the cohort index was set from January 2007 to December 2011 and the follow-up period was set from January 2007 to December 2013 (the end of the NHIS-NSC data), or the expired date of the patients (Figure 1).

### Subjects

**FIGURE 2:**
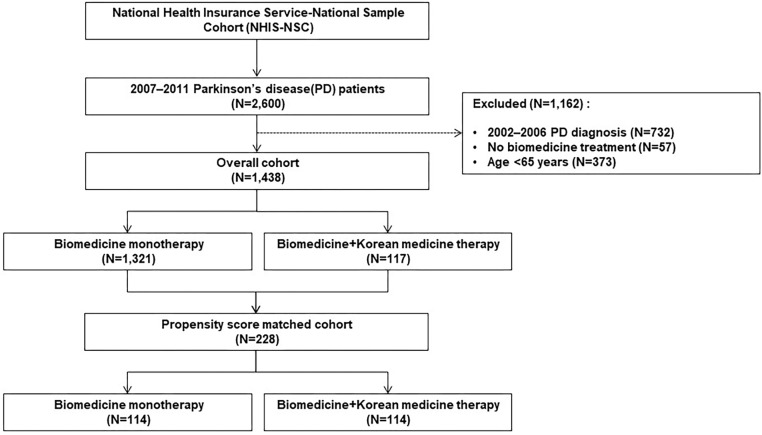
Selection of subjects.

From 2007 to 2011, a cohort of patients aged 65 years or older who were diagnosed with PD by the NHIS-NSC was established. The diagnosis was defined by the G20 code based on the Korean Standard Classification of Diseases (KCD), which is the same as the 10^th^ revision of the International Classification of Diseases (ICD-10). Patients who were diagnosed with PD during the eligibility period were excluded; therefore, only those newly diagnosed with PD from 2007 to 2011 were included (Figure 1). Patients were divided into two groups by treatment type, an integrative therapy group that received both biomedicine and KM, and a monotherapy group that was only treated with biomedicine. When treatment history for PD was recorded at least twice in each form, the patient was considered to have received the treatment. However, patients who received the treatment only once were excluded (Figure 2).

### Outcome Variables

Generally, the outcome of PD recovery or management is based on the UPDRS, SCOPA, or the PDQ-39 (Peto et al., 1995; Marinus et al., 2004; Goetz et al., 2008). However, the NHIS-NSC does not have survey or examination data such as health insurance claims data. Thus, this study used alternative outcomes to demonstrate that PD and its complications had been well-controlled through alternative outcomes. The outcome was defined as the prevalence of hospitalization and annual length of hospitalization after PD diagnosis. Hospitalization was classified as total hospitalization, hospitalization because of PD and hospitalization due to major complications of PD. In this study, dementia, depression, and pneumonia/sepsis were considered major complications. KCD F00–F09, G30–G32 for dementia, F30–F39 and U22 for depression, and A40–A41, J12–J18, J69 and J80–J84 for pneumonia/sepsis were applied.

### Statistical Analysis

Confounders were controlled for comparison between the integrative therapy group and the monotherapy group. In this study, the CCI was calculated using the KCD code of the NHIS-NSC and had a possible score of 0 to 12 points. The CCI gives weight to several comorbidity factors that affect death, and has been mapped to ICD codes through clinical review (Quan et al., 2005). In addition, the severity of disability of the patients included in the NHIS-NSC was considered as a confounder. To adjust for biomedicine treatment in both treatment groups, the treatment period in neurology, hospitalization period in neurology and anti-parkinsonism medications (levodopa, ropinirole, pramipexole, selegiline) were also considered as confounders (Zhuo et al., 2017).

The propensity score (PS) was used to increase the level of causality revealed in the observational study (D’Agostino, 1998). Specifically, the PS was used to match the subjects with similar characteristics in the two groups. Several variables were used to create the PS index: age, sex, index year, CCI, treatment period in neurology, hospitalization period in neurology, severity of disability and anti-parkinsonism medication. Comparison of the PS between groups was repeated using the greedy algorithm and 1: 1 matching continued until there were no more matching pairs (Parsons, 2001). The PS fit was confirmed by c-statistics and the Hosmer and Lemeshow Goodness-of-Fit test. In the case of a c-statistic value over 0.5, the Hosmer and Lemeshow Goodness-of-Fit test was considered to be good when the *p*-value was 0.05 or higher (D’Agostino, 1998; Maldonado and Greenland, 2002).

The prevalence and annual length of hospitalization were analyzed using logistic regression analysis and *t*-tests. A *p*-value < 0.05 was considered significant. Additionally, the integrative therapy group was divided into two groups by treatment period, less than 30 days and over 30 days. The prevalence and annual length of hospitalization were analyzed using logistic regression analysis and ANOVA. After ANOVA, Duncan’s *post hoc* test was applied. A *p*-value < 0.05 was considered significant. Finally, survival analysis of the integrative therapy group and monotherapy group was performed using the Cox proportional hazard analysis.

All statistical analyses were performed using SAS 9.4.

## Results

### Characteristics of Patients

Except for patients diagnosed with PD prior to 2007, patients treated without biomedicine treatment and those younger than 65 years, a total of 1,438 patients were included in the cohort. Of these, 117 were treated using both biomedicine and Korean medicine, while 1,321 received biomedicine alone. After PS estimation and matching, the cohort used in the analysis included 228 subjects (114 in the integrative therapy group and 114 in the monotherapy group) (Figure 2). The PS matching was considered to be well-fitted based on a c-statistics value of 0.810 and a *p*-value of the Goodness-of-Fit Test of 0.141.

The patients’ characteristics based on confounders in the two groups before and after PS matching were compared. After PS matching, the characteristics of both groups were statistically similar (Table 1).

**Table 1:** Comparison of characteristics between integrative therapy group and monotherapy group.

	Before PS matching			After PS matching		
	Integrative therapy group (*N* = 117)	Monotherapy group (*N* = 1,321)	*p*-Value	Integrative therapy group (*N* = 114)	Monotherapy Group (*N* = 114)	*p*-Value
**Sex**			0.512			0.487
Male	38 (32.5%)	469 (35.5%)		37 (32.5%)	42 (36.8%)	
Female	79 (67.5%)	852 (64.5%)		77 (67.5%)	72 (63.2%)	
**Age**			<0.001*			0.686
65–69	37 (31.6%)	241 (18.2%)		34 (29.8%)	28 (24.6%)	
70–74	30 (25.6%)	374 (28.3%)		30 (26.3%)	27 (23.7%)	
75–79	35 (29.9%)	346 (26.2%)		35 (30.7%)	41 (36.0%)	
=80	15 (12.8%)	360 (27.3%)		15 (13.2%)	18 (15.8%)	
**Index year**			0.016*			0.491
2007	7 (6.0%)	225 (17.0%)		7 (6.1%)	5 (4.4%)	
2008	23 (19.7%)	279 (21.1%)		23 (20.2%)	23 (20.2%)	
2009	25 (21.4%)	278 (21.0%)		25 (21.9%)	16 (14.0%)	
2010	26 (22.2%)	236 (17.9%)		24 (21.1%)	26 (22.8%)	
2011	36 (30.8%)	393 (22.9%)		35 (30.7%)	44 (38.6%)	
**Charlson Comorbidity Index**			0.308			0.489
0	0 (0.0%)	5 (0.4%)		0 (0.0%)	0 (0.0%)	
1	17 (14.5%)	290 (22.0%)		17 (14.9%)	21 (18.4%)	
3	88 (75.2%)	900 (68.1%)		86 (75.4%)	87 (76.3%)	
4	2 (1.7%)	8 (0.6%)		1 (0.9%)	0 (0.0%)	
6	10 (8.6%)	117 (8.9%)		10 (8.8%)	6 (5.3%)	
9	0 (0.0%)	1 (0.1%)		0 (0.0%)	0 (0.0%)	
**Treatment period in neurology (days)**			<0.001*			0.992
≤29	19 (16.2%)	664 (50.3%)		19 (16.7%)	18 (15.8%)	
30–179	29 (24.8%)	328 (24.8%)		29 (25.4%)	30 (26.3%)	
180–364	12 (10.3%)	82 (6.2%)		12 (10.5%)	11 (9.7%)	
≥365	57 (48.7%)	247 (18.7%)		54 (47.4%)	55 (48.3%)	
**Hospitalization period in neurology (days)**			<0.001*			0.966
≤13	15 (12.8%)	616 (46.6%)		15 (13.2%)	15 (13.2%)	
14–29	26 (22.2%)	267 (20.2%)		25 (21.9%)	22 (19.3%)	
30–59	17 (14.5%)	208 (15.8%)		17 (14.9%)	17 (14.9%)	
>60	59 (50.4%)	230 (17.4%)		57 (50.0%)	60 (52.6%)	
**Severity of disability**			0.477			0.729
Normal	89 (76.1%)	1,005 (76.1%)		86 (75.4%)	91 (79.8%)	
Mild	22 (18.8%)	213 (16.1%)		22 (19.3%)	18 (15.8%)	
Severe	6 (5.1%)	103 (7.8%)		6 (5.3%)	5 (4.4%)	
**Anti-parkinsonism medication**						
Levodopa	68 (58.1%)	326 (24.7%)	<0.001*	65 (57.0%)	58 (50.9%)	0.352
Ropinirole	34 (29.1%)	126 (9.5%)	<0.001*	32 (28.1%)	27 (23.7%)	0.450
Pramipexole	18 (15.4%)	74 (5.6%)	<0.001*	16 (14.0%)	15 (13.2%)	0.847
Selegiline	5 (4.3%)	26 (2.0%)	0.100	5 (4.4%)	3 (2.6%)	0.472


*PS, propensity score; monotherapy group, treated by biomedicine only; integrative therapy group, treated by biomedicine and KM. ^∗^Statistically significant (*p* < 0.05).*

### Types of KM Used in the Integrative Group

**FIGURE 3:**
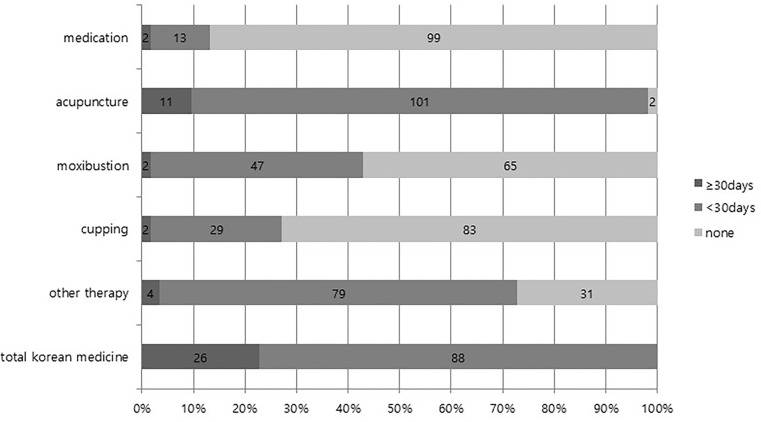
Status of KM therapy in integrative therapy group.

Among 114 patients in the integrative group, 112 patients (98.2%) received acupuncture therapy at least two times, 49 (43.0%) received moxibustion therapy, 31 (27.2%) received cupping therapy, 15 (13.2%) received herbal medication, and 83 (72.8%) received other physical therapy or psychotherapy. The results of dividing the number of days on which KM was applied by 30 days are presented in Figure 3.

### Comparison of Prevalence of Hospitalization

Comparison of the prevalence of hospitalization between the two groups was performed by logistic regression analysis, after which the odds ratio was calculated. The prevalence of hospitalization because of pneumonia/sepsis was 0.50 (95% C.I.: 0.26–0.96) times lower in the integrative therapy group than the monotherapy group (*p* = 0.038). The prevalence of total hospitalization, as well as hospitalization because of PD, dementia, and depression in the integrative therapy group was positive compared to the monotherapy group, but this difference was not significant.

Additionally, the prevalence of total hospitalization, hospitalization because of PD, dementia, depression, and pneumonia/sepsis showed positive results in the order of integrative group II (KM 30 days or more), integrative group I (KM less than 30 days), monotherapy group, but this difference was not significant (Table 2).

**Table 2:** Comparison of prevalence of hospitalization between integrative therapy group and monotherapy group (unit: no. of subjects).

	No. of subjects (%)	OR (95% C.I.)	*p*-Value
**Total hospitalization**			
Monotherapy group (*N* = 114)	103 (90.4%)	1	
Integrative therapy group (*N* = 114)	99 (86.8%)	0.71 (0.31–1.61)	0.406
Monotherapy group (*N* = 114)	103 (90.4%)	1	
Integrative therapy group I (*N* = 88)	77 (87.5%)	0.75 (0.31–1.81)	0.520
Integrative therapy group II (*N* = 26)	22 (84.6%)	0.59 (0.17–2.02)	0.398
**Hospitalization d/t PD**			
Monotherapy group (*N* = 114)	79 (69.3%)	1	
Integrative therapy group (*N* = 114)	74 (64.9%)	0.82 (0.47–1.43)	0.481
Monotherapy group (*N* = 114)	79 (69.3%)	1	
Integrative therapy group I (*N* = 88)	61 (69.3%)	1.00 (0.55–1.83)	0.998
Integrative therapy group II (*N* ( =26)	13 (50.0%)	0.44 (0.19–1.05)	0.065
**Hospitalization d/t dementia**			
Monotherapy group (*N* = 114)	19 (16.7%)	1	
Integrative therapy group (*N* = 114)	10 (8.8%)	0.48 (0.21–1.09)	0.078
Monotherapy group (*N* = 114)	19 (16.7%)	1	
Integrative therapy group I (*N* = 88)	9 (10.2%)	0.57 (0.24–1.33)	0.193
Integrative therapy group II (*N* = 26)	1 (3.8%)	0.20 (0.03–1.57)	0.125
**Hospitalization d/t depression**			
Monotherapy group (*N* = 114)	5 (4.4%)	1	
Integrative therapy group (*N* = 114)	1 (0.9%)	0.19 (0.02–1.68)	0.136
Monotherapy group (*N* = 114)	5 (4.4%)	1	
Integrative therapy group I (*N* = 88)	1 (1.1%)	0.25 (0.03–2.19)	0.242
Integrative therapy group II (*N* = 26)	0 (0%)	-	-
**Hospitalization d/t pneumonia/sepsis**			
Monotherapy group (*N* = 114)	31 (27.2%)	1	
Integrative therapy group (*N* = 114)	18 (15.8%)	0.50 (0.26–0.96)	0.038*
Monotherapy group (*N* = 114)	31 (27.2%)	1	
Integrative therapy group I (*N* = 88)	15 (17.0%)	0.55 (0.28–1.10)	0.091
Integrative therapy group II (*N* = 26)	3 (11.5%)	0.35 (0.10–1.25)	0.105


*OR, odds ratio; C.I., confidence interval; monotherapy group, treated by biomedicine only; integrative therapy group, treated by biomedicine and KM; integrative therapy group I, KM treatment less than 30 days; integrative therapy group II, KM treatment over than 30 days; d/t, due to; PD, Parkinson’s disease. ^∗^Statistically significant (*p* < 0.05)*.

### Comparison of Annual Length of Hospitalization

**FIGURE 4:**
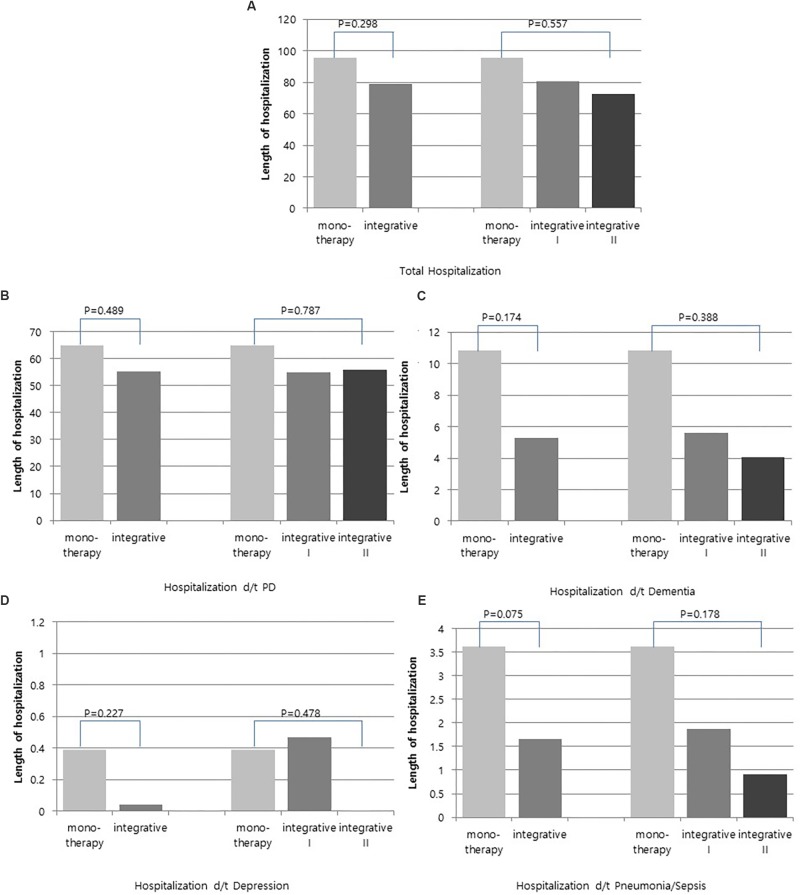
Comparison of annual length of hospitalization between integrative therapy group and monotherapy group. **(A)** Total hospitalization, **(B)** Hospitalization d/t PD, **(C)** Hospitalization d/t dementia, **(D)** Hospitalization d/t depression, and **(E)** Hospitalization d/t pneumonia/sepsis.

Comparison of the annual length of hospitalization between groups using a *t*-test revealed that total annual length of hospitalization, annual length of hospitalization due to PD, annual length of hospitalization due to dementia, annual length of hospitalization due to depression and annual length of hospitalization due to pneumonia/sepsis were shorter in the integrative therapy group than the monotherapy group; however, these differences were not statistically significant. The integrative therapy group was divided into two groups (over 30 days, less than 30 days) by treatment duration, and ANOVA was conducted to compare each of these with the monotherapy group. The prevalence of total hospitalization, as well as hospitalization because of PD, dementia, depression and pneumonia/sepsis showed positive results in the order of integrative group II (KM 30 days or more), integrative group I (KM less than 30 days), and monotherapy group, but these differences were not significant (Figure 4).

The cox proportional hazard model was used to compare the mortality between groups. The mortality of the integrative therapy group was found to be 4.51 patients per 1,000 person-years, while that of the monotherapy group was 9.21 patients per 1,000 person-years and the hazard ratio (HR) was 0.50. However, these differences were not significant.

## Discussion

The most typical medication for PD is levodopa; however, its long-term use results in decreased efficacy, motor fluctuation, and dyskinesia in about 75% of patients (Fahn et al., 2004). The prevalence of melanoma in PD patients taking levodopa is significantly higher than that of the general population (Shalaby and Louis, 2016). Dementia in PD patients is a common complication of PD that makes treatment difficult and leads to disability. Depression is also a common complication of PD (Brown and Marsden, 1984; Cummings, 1992), and infectious diseases such as pneumonia are considered factors that increase mortality in PD patients (Pinter et al., 2015). Additionally, patients with a longer duration of PD have more complications and lower quality of life (Ou et al., 2018). Subthalamic stimulation has been attempted to improve quality of life with positive results. However, surgery can be accompanied by unintended adverse events, even death (Dafsari et al., 2018). Therefore, the use of relatively safe treatment regimens such as CAM should be considered.

This study investigated whether KM could reduce hospitalization in the management of PD patients in the South Korea. Two groups of patients, an integrative therapy group using both biomedicine and KM and a monotherapy group using only biomedicine, were compared by PS estimation and matching based on confounders such as sex, age, comorbidity, and anti-parkinsonism medication. The prevalence of hospitalization because of pneumonia/sepsis was significantly lower (0.50 times) in the integrative therapy group than the monotherapy group. Traditional medicine may have beneficial immunomodulatory effects for preventing viral infections (Poon et al., 2006). For severe acute respiratory syndrome (SARS), advantages to treatment with integrative Chinese medicine and biomedicine were observed when compared with that of biomedicine alone, especially with respect to improvement of clinical symptoms, promotion of the recovery of immune function and reduction of treatment duration (Qiang et al., 2003). Hence, KM seems to have a positive effect on infectious diseases such as pneumonia and sepsis.

Other outcomes of the integrative therapy group also showed better results than the monotherapy group, but these differences were not significant. Although there were no significant differences in the outcomes observed in this study, the prevalence and duration of hospitalization tended to be lower in patients who received KM. Specifically, a KM treatment period of 30 days or more showed better results than that of less than 30 days.

Studies have shown that acupuncture is effective at the treatment of PD (Lee and Lim, 2017). For example, PD patients with acupuncture showed significant improvement in motor function compared to those without acupuncture. Moreover, brain imaging revealed that putamen and the primary motor cortex were activated in patients with acupuncture (Chae et al., 2009). Korean herbal formulas such as Chungsimyeolda-tang or Yeoldahanso-tang induce autophagy and prevent diseases associated with misfolded/aggregated proteins in various neurodegenerative disorders, including PD (Bae et al., 2011, 2015). In China, 48.6% of patients underwent CAM treatment and herbal medication, primarily rehabilitation exercise and acupuncture. These treatments were effective in half of the patients who received them, while they produced negative results in 11% of the patients (Pan et al., 2018). Additionally, the incidence and prevalence of PD in Asian countries, including South Korea, have been shown to be lower than in Europe and America (Pringsheim et al., 2014). This difference may result from genetic and ethnic differences, but the possibility of the effects of CAM, including KM, cannot be ruled out.

It should be noted that this paper had several limitations. First, the number of subjects was not sufficient because of data limitations; therefore, we cannot be sure of whether the non-significance of results is due to the absence of a relationship between the groups or to a lack of statistical power. Accordingly, comparative studies with increased duration and number of patients are required to clarify the effects KM on PD. Second, we assessed whether PD and its complications have been well-controlled by alternative outcomes-related measures such as the prevalence of hospitalization and annual length of hospitalization after PD diagnosis. Generally, the outcome of PD recovery or management is evaluated based on the UPDRS, SCOPA, or the PDQ-39. However, the NHIS-NSC does not contain this information because it consists of only health insurance claim data. Third, the number of KM monotherapy patients was very small. In the Korean health insurance system, PD is a serious rare disease that is registered and managed by the government. After PD patients are confirmed and registered 90% of the cost of treatment is supported by the government. In addition to receiving standard drug therapies such as levodopa, registered patients can also concurrently use KM such as herbal formulas and acupuncture if they desire. Therefore, further study is necessary to analyze the total national insurance dataset because the NHIS-NSC, which was used in our study, is equivalent to only 2.2% of the population of the South Korea.

## Conclusion

It has not been clearly identified that integrative therapy with KM and biomedicine for PD management is better treatment for patients compared to biomedicine monotherapy; however, we found a clue of better result in integrated therapy. However, it should be noted that further investigation by increasing the number of subjects is needed.

## Ethics Statement

This study was approved by the Institutional Review Board of Dongguk University, Gyeongju (DRG IRB 20170015). Patient consent was exempted because of the total anonymity of all research data used in this study.

## Author Contributions

YW and MH planned the study and wrote the manuscript.

## Conflict of Interest Statement

The authors declare that the research was conducted in the absence of any commercial or financial relationships that could be construed as a potential conflict of interest.

## Abbreviations

CCI: Charlson Comorbidity Index
KM: Korean medicine
NHIS-NSC: National Health Insurance Service-National Sample Cohort
PD: Parkinson’s disease
PDQ-39: Parkinson’s Disease Questionnaire
SCOPA: Scales for Outcomes in Parkinson’s Disease
UPDRS: Unified Parkinson’s Disease Rating Scale

## References

[B1] Ascherio A. Schwarzschild M. A. (2016). The epidemiology of Parkinson’s disease: risk factors and prevention. *Lancet Neurol.* 15 1257–1272. 10.1016/S1474-4422(16)30230-7 27751556

[B2] Bae N. Ahn T. Chung S. Oh M. S. Ko H. Oh H. (2011). The neuroprotective effect of modified Yeoldahanso-tang via autophagy enhancement in models of Parkinson’s disease. *J. Ethnopharmacol.* 134 313–322. 10.1016/j.jep.2010.12.016 21172413

[B3] Bae N. Chung S. Kim H. J. Cha J. W. Oh H. Gu M.-Y. (2015). Neuroprotective effect of modified Chungsimyeolda-tang, a traditional Korean herbal formula, via autophagy induction in models of Parkinson×s disease. *J. Ethnopharmacol.* 159 93–101. 10.1016/j.jep.2014.11.007 25449460

[B4] Bega D. Zadikoff C. (2014). Complementary & alternative management of Parkinson’s disease: an evidence-based review of eastern influenced practices. *J. Mov. Disord.* 7 57–66. 10.14802/jmd.14009 25360229 PMC4213533

[B5] Brown R. G. Marsden C. D. (1984). How common is dementia in Parkinson’s disease? *Lancet* 2 1262–1265. 6150288 10.1016/s0140-6736(84)92807-1

[B6] Chae Y. Lee H. Kim H. Kim C.-H. Chang D.-I. Kim K.-M. (2009). Parsing brain activity associated with acupuncture treatment in parkinson’s diseases. *Mov. Disord.* 24 1794–1802. 10.1002/mds.22673 19533753

[B7] Cummings J. L. (1992). Depression and parkinson’s disease: a review. *Am. J. Psychiatry* 149 443–454. 10.1176/ajp.149.4.443 1372794

[B8] Cwiekala-Lewis K. J. Gallek M. Taylor-Piliae R. E. (2017). The effects of Tai Chi on physical function and well-being among persons with parkinson’s disease: a systematic review. *J. Bodyw. Mov. Ther.* 21 414–421. 10.1016/j.jbmt.2016.06.007 28532886

[B9] Dafsari H. S. Reker P. Stalinski L. Silverdale M. Rizos A. Ashkan K. (2018). Quality of life outcome after subthalamic stimulation in parkinson’s disease depends on age. *Mov. Disord.* 33 99–107. 10.1002/mds.27222 29150860

[B10] D’Agostino R. B. Jr. (1998). Propensity score methods for bias reduction in the comparison of a treatment to a non-randomized control group. *Stat. Med.* 17 2265–2281. 10.1002/(SICI)1097-0258(19981015)17:19<2265::AID-SIM918>3.0.CO;2-B 9802183

[B11] Delamarre A. Meissner W. G. (2017). Epidemiology, environmental risk factors and genetics of parkinson’s disease. *Presse Med.* 46 175–181. 10.1016/j.lpm.2017.01.001 28189372

[B12] Elbaz A. Carcaillon L. Kab S. Moisan F. (2016). Epidemiology of Parkinson’s disease. *Rev. Neurol.* 172 14–26. 10.1016/j.neurol.2015.09.012 26718594

[B13] Fahn S. Oakes D. Shoulson I. Kieburtz K. Rudolph A. Lang A. (2004). Levodopa and the progression of parkinson’s disease. *N. Engl. J. Med.* 351 2498–2508. 10.1056/NEJMoa033447 15590952

[B14] Ghaffari B. D. Kluger B. (2014). Mechanisms for alternative treatments in parkinson’s disease: acupuncture, tai chi, and other treatments. *Curr. Neurol. Neurosci. Rep.* 14:451. 10.1007/s11910-014-0451-y 24760476

[B15] Goetz C. G. Tilley B. C. Shaftman S. R. Stebbins G. T. Fahn S. Martinez-Martin P. (2008). Movement Disorder Society-sponsored revision of the Unified Parkinson’s Disease Rating Scale (MDS-UPDRS): scale presentation and clinimetric testing results. *Mov. Disord.* 23 2129–2170. 10.1002/mds.22340 19025984

[B16] Jang J. H. Lee J. Jung I. Yoo H. (2018). Efficacy of Yokukansankachimpihange on sleep disturbance in Parkinson’s disease: a study protocol of a randomized, double blind, placebo-controlled pilot trial. *Medicine* 97:e11298. 10.1097/MD.0000000000011298 29953013 PMC6039679

[B17] Lee H. J. Kim S. Y. Chae Y. Kim M. Y. Yin C. Jung W. S. (2018). Turo (Qi Dance) program for parkinson’s disease patients: randomized, assessor blind, waiting-list control, partial crossover study. *Explore* 14 216–223. 10.1016/j.explore.2017.11.002 29650371

[B18] Lee J. Lee J. S. Park S. H. Shin S. A. Kim K. (2017). Cohort profile: the National Health Insurance Service-National Sample Cohort (NHIS-NSC), South Korea. *Int. J. Epidemiol.* 46:e15. 26822938 10.1093/ije/dyv319

[B19] Lee S. H. Lim S. (2017). Clinical effectiveness of acupuncture on Parkinson disease: a PRISMA-compliant systematic review and meta-analysis. *Medicine* 96:e5836. 10.1097/MD.0000000000005836 28099340 PMC5279085

[B20] Leem J. (2016). Acupuncture for motor symptom improvement in parkinson’s disease and the potential identification of responders to acupuncture treatment. *Integr. Med. Res.* 5 332–335. 10.1016/j.imr.2016.06.006 28462136 PMC5390418

[B21] Maldonado G. Greenland S. (2002). Estimating causal effects. *Int. J. Epidemiol.* 31 422–429. 10.1093/intjepid/31.2.422 11980807

[B22] Marinus J. Visser M. Stiggelbout A. M. Rabey J. M. Martínez-Martín P. Bonuccelli U. (2004). A short scale for the assessment of motor impairments and disabilities in parkinson’s disease: the SPES/SCOPA. *J. Neurol. Neurosurg. Psychiatry* 75 388–395. 10.1136/jnnp.2003.017509 14966153 PMC1738938

[B23] Noh H. Kwon S. Cho S. Y. Jung W. S. Moon S. K. Park J. M. (2017). Effectiveness and safety of acupuncture in the treatment of parkinson’s disease: a systematic review and meta-analysis of randomized controlled trials. *Complement. Ther. Med.* 34 86–103. 10.1016/j.ctim.2017.08.005 28917379

[B24] Ou R. Hou Y. Song W. Wei Q. Chen Y. Cao B. (2018). Clinical characteristics and quality of life in Chinese patients with parkinson’s disease beyond 20 years. *Neurol. Res.* 40 312–317. 10.1080/01616412.2018.1438227 29447582

[B25] Pan X.-W. Zhang X.-G. Chen X.-C. Lu Q. Hu Y.-S. Han L.-Y. (2018). A survey of application of complementary and alternative medicine in chinese patients with parkinson’s disease: a pilot study. *Chin. J. Integr. Med.* 10.1007/s11655-018-2560-y [Epub ahead of print]. 29915907

[B26] Parsons L. (2001). “Reducing bias in a propensity score matched-pair sample using greedy matching techniques,” in *Proceedings of the 26th Annual SAS Users Group International Conference* (Long Beach, CA) 214–226.

[B27] Peto V. Jenkinson C. Fitzpatrick R. Greenhall R. (1995). The development and validation of a short measure of functioning and well being for individuals with Parkinson’s disease. *Qual. Life Res.* 4 241–248. 10.1007/BF02260863 7613534

[B28] Pinter B. Diem-Zangerl A. Wenning G. K. Scherfler C. Oberaigner W. Seppi K. (2015). Mortality in parkinson’s disease: a 38-year follow-up study. *Mov. Disord.* 30 266–269. 10.1002/mds.26060 25447933

[B29] Poon P. M. K. Wong C. K. Fung K. P. Fong C. Y. S. Wong E. L. Y. Lau J. T. F. (2006). Immunomodulatory effects of a traditional chinese medicine with potential antiviral activity: a self-control study. *Am. J. Chin. Med.* 34 13–21. 10.1142/S0192415X0600359X 16437735

[B30] Pringsheim T. Jette N. Frolkis A. Steeves T. D. (2014). The prevalence of parkinson’s disease: a systematic review and meta-analysis. *Mov. Disord.* 29 1583–1590. 10.1002/mds.25945 24976103

[B31] Qiang J. Biao W. Rui-Lin Z. Bao-Guo W. Li-Min F. Hai-Jian W. (2003). Clinical controlled study of integrative chinese and western medicine in treating 49 cases of SARS. *Chin. J. Integr. Med.* 9 175–180. 10.1007/BF02838027

[B32] Quan H. Sundararajan V. Halfon P. Fong A. Burnand B. Luthi J. C. (2005). Coding algorithms for defining comorbidities in ICD-9-CM and ICD-10 administrative data. *Med. Care* 43 1130–1139. 10.1097/01.mlr.0000182534.19832.83 16224307

[B33] Rizek P. Kumar N. Jog M. S. (2016). An update on the diagnosis and treatment of parkinson disease. *CMAJ* 188 1157–1165. 10.1503/cmaj.151179 27221269 PMC5088077

[B34] Rogers G. Davies D. Pink J. Cooper P. (2017). Parkinson’s disease: summary of updated NICE guidance. *BMJ* 358:j1951. 10.1136/bmj.j1951 28751362

[B35] Shalaby S. Y. Louis E. D. (2016). Increased odds of melanoma: parkinson’s disease, essential tremor, dystonia versus controls. *Neuroepidemiology* 46 128–136. 10.1159/000443794 26820576 PMC5473151

[B36] Shan C. S. Zhang H. F. Xu Q. Q. Shi Y. H. Wang Y. Li Y. (2018). Herbal medicine formulas for parkinson’s disease: a systematic review and meta-analysis of randomized double-blind placebo-controlled clinical trials. *Front. Aging Neurosci.* 10:349 10.3389/fnagi.2018.00349 PMC6236206 30467472

[B37] Yang S.-B. Kim Y.-J. Lee H.-M. Lee H.-J. Cho S.-Y. Park J.-M. (2016). Effects of korean medicine on patients with idiopathic parkinson’s disease: a retrospective study. *J. Intern. Korean Med.* 37 653–660. 10.22246/jikm.2016.37.4.653 28753030

[B38] Zhuo C. Zhu X. Jiang R. Ji F. Su Z. Xue R. (2017). Comparison for efficacy and tolerability among ten drugs for treatment of parkinson’s disease: a network meta-analysis. *Sci. Rep.* 8:45865. 10.1038/srep45865 28374775 PMC5379205

